# Potential Benefits of TNF Targeting Therapy in Blau Syndrome, a NOD2-Associated Systemic Autoinflammatory Granulomatosis

**DOI:** 10.3389/fimmu.2022.895765

**Published:** 2022-05-27

**Authors:** Tomoko Matsuda, Naotomo Kambe, Riko Takimoto-Ito, Yoko Ueki, Satoshi Nakamizo, Megumu K. Saito, Syuji Takei, Nobuo Kanazawa

**Affiliations:** ^1^ Department of Dermatology, Kansai Medical University, Hirakata, Japan; ^2^ Department of Dermatology, Kyoto University Graduate School of Medicine, Kyoto, Japan; ^3^ Department of Clinical Application, Center for iPS Cell Research and Application, Kyoto University, Kyoto, Japan; ^4^ Department of Pediatrics, Kagoshima University, Kagoshima, Japan; ^5^ Department of Dermatology, Hyogo Medical University, Nishinomiya, Japan

**Keywords:** Blau syndrome, NOD2, granuloma, IFNγ, TNF

## Abstract

Blau syndrome is a systemic autoinflammatory granulomatous disease caused by mutations in the nucleotide-binding oligomerization domain 2 (*NOD2*) gene. NOD2 is an intracellular pathogen recognition receptor. Upon binding to muramyl dipeptide (MDP), NOD2 activates the NF-κB pathway, leading to the upregulation of proinflammatory cytokines. Clinical manifestations of Blau syndrome appear in patients before the age of four. Skin manifestations resolve spontaneously in some cases; however, joint and eye manifestations are progressive, and lead to serious complications, such as joint contracture and blindness. Currently, there is no specific curative treatment for the disease. Administration of high-dose oral steroids can improve clinical manifestations; however, treatments is difficult to maintain due to the severity of the side effects, especially in children. While several new therapies have been reported, including JAK inhibitors, anti-IL-6 and anti-IL-1 therapies, anti-TNF therapy plays a central role in the treatment of Blau syndrome. We recently performed an *ex vivo* study, using peripheral blood and induced pluripotent stem cells from patients. This study demonstrated that abnormal cytokine expression in macrophages from untreated patients requires IFNγ stimulation, and that anti-TNF treatment corrects the abnormalities associated with Blau syndrome, even in the presence of IFNγ. Therefore, although the molecular mechanisms by which the genetic mutations in *NOD2* lead to granuloma formation remain unclear, it is possible that prior exposure to TNFα combined with IFNγ stimulation may provide the impetus for the clinical manifestations of Blau syndrome.

## Introduction

Blau syndrome (MIM #186580) is a rare, systemic granulomatous disease caused by mutations in the nucleotide-binding oligomerization domain 2 (*NOD2*) gene and is inherited in an autosomal dominant manner. It is classified as an autoinflammatory syndrome, a concept that has received increasing attention in recent years.

In 1985, Blau (1) reported a family that caused granulomas in the skin, eyes, and joints over four generations. In 1990, Pastores et al. (2) reported a mother and daughter with similar symptoms and considered them to be from the same category as the diseases reported by Blau; thus, they named the disease Blau syndrome. Subsequently, a linkage analyses of a large pedigree were presumed, and discovered that Blau syndrome and Crohn’s disease have mutations in the same gene (3). In 2001, when mutations in the *NOD2* gene were identified in Crohn’s disease (4, 5), Miceli-Richard et al. (6) examined the *NOD2* gene in four families with Blau syndrome, and identified three gene mutations.

Idiopathic sarcoidosis pediatric cases are rare, yet it is known that in a small subset of patients, peak onset occurs before the age of four years (less than 0.5% of all sarcoidosis cases) (7). The clinical features in these cases were the absence of bilateral hilar lymphadenopathy, and the presence of joint symptoms. Hence, these cases were occasionally referred to as early-onset sarcoidosis (EOS), and the difference from idiopathic sarcoidosis debated (8). Initially no gene abnormalities were found in two solitary cases of EOS (6). We collected ten EOS cases in Japan and identified the same *NOD2* mutations in these patients as those reported for Blau syndrome (9), and now Blau syndrome and EOS are considered to be the same disease (10, 11).

## Molecular Mechanisms

NOD2 consists of 1,040 amino acids and has a three domain structure. The N-terminus contains two caspase activation and recruitment domain s (CARD), which are important as in signal transduction; the centrally located NOD region is involved in polymerization; and the C-terminus contains a leucine-rich repeat (LRR). The LRR recognizes muramyl dipeptide (MDP), a component of bacterial cell walls. In Blau syndrome, most patients have mutations in exon 4, the NOD region. Most of these mutations are missense, with single amino acid substitutions. The 334th amino acid of NOD2 is a hot spot for mutations. Typically, arginine (R) is mutated to glutamine (Q) or tryptophan (W) in this region (p.R334Q and p.R334W). Among the 50 patients with *NOD2* mutations currently identified in Japan, the p.R334W mutation was most common (fifteen cases), followed by the p.R587C (nine cases), and then the p.R334Q (five cases) (12).

It is still unclear how the mutations identified in Blau syndrome are involved in the formation of granulomas. When NOD2 was shown to recognize MDP, an experimental system to overexpress the NOD2 genes into HEK293 cells was used, in which the mutant with a frameshift in the LRR frequently identified in Crohn’s disease was shown to be hyporesponsive to MDP (13). In contrast, mutants identified in Blau syndrome spontaneously promoted NF-κB transcription in the same experimental system, even without the addition of MDP (14). This system of assessing NF-κB transcriptional capacity using luciferase as an indicator is still today a very useful system for confirming whether identified mutations are those associated with Blau syndrome, and indeed all 15 mutants we identified showed spontaneous NF-κB transcriptional enhancement in the absence of MDP (9, 15). This ligand-independent NF-κB activation induced by mutations has been observed in another group of hereditary autoinflammatory syndromes known as cryopyrin-associated periodic syndromes (CAPS). CAPS is caused by mutations in the *NLRP3* gene, another NOD family receptor (16). Interestingly, the p.R334W mutation in the *NOD2* gene frequently found in Blau syndrome, corresponds to a missense mutation in an analogous position of the *NLRP3* gene, the p.R260W mutation in CAPS. These mutations suggest a common molecular mechanism, inducing the autoactivation of NOD family receptors, between these two inflammatory diseases (17).

Nevertheless, we observe an overproduction of IL-1β in peripheral blood monocytes extracted from CAPS patients and the macrophages established from CAPS patients-derived induced pluripotent stem (iPS) cells compared to healthy controls (18, 19), whereas a reduced response to MDP to produce inflammatory cytokines in the peripheral blood of patients with Blau syndrome were reported (20, 21). This lower responsiveness to MDP at the cellular level was also observed in studies using iPS cells differentiated into macrophages established from our patients (22), or knock-in mice with a corresponding *Nod2* mutation identified in Blau syndrome (23). Since granulomas may arise from the inability to eliminate foreign substances and pathogens, it is possible that a reduced response to MDP at the cellular level may induce chronic inflammation, leading to granuloma formation in Blau syndrome.

## Clinical Manifestations

The clinical phenotype of Blau syndrome is characterized by a distinct triad of skin, joint and eye disorders.

### Skin Rash

Skin lesions often present as a first symptom (12). The most frequent skin manifestations are scaly erythematous plaques with multiple lichenoid papules and no apparent subjective symptoms. Occasionally, a BCG vaccination is the trigger for a skin rash that closely resembles lichen scrofulous, a tuberculous rash. Histological findings are characterized by the presence of epithelioid granulomas, with giant cells in the dermis. Cutaneous manifestations may disappear spontaneously and are often overlooked without a proper diagnosis, due to the lack of subjective symptoms. Cases of erythema nodosum have also been reported (15).

### Joint Symptoms

The skin rash is followed by joint symptoms (12). Symmetrical polyarthritis occurs in small joints, such as the fingers and toes; and large joints, such as the hands, elbows, knees, feet; and rarely in the shoulders (24, 25). Painless, cystic swelling of the dorsal surfaces of the wrist and ankle, sausage-like swelling of the fingers and toes especially toward the base, and camptodactyly are characteristic findings.

The absence of arthralgia in the presence of joint swelling, the lack of an initial limitation of movement, and the presence of cystic swelling on the dorsum of the hands and feet, without pain, are of high diagnostic value and are important in differentiating the disease from juvenile idiopathic arthritis (JIA). In Blau syndrome, inflammation of the joint synovium is rare, and in the early stages of the disease, the tendon sheath synovium is damaged, leading to edema around the synovium and a limitation of movement. Joint sonography (26, 27) and MRI are useful to identify the main site of inflammation. However, in children, it is difficult to distinguish synovial thickening due to the lack of ossification in the cartilage on the surface of the bone, and a large area of low-density echoes.

### Ocular Manifestations

Ocular manifestations appear later than skin and joint symptoms (12). The most common ocular manifestation is a bilateral uveitis (28–30). Other symptoms include posterior iris adhesion, conjunctivitis, retinitis, and optic nerve atrophy, all of which affect the entire eye. If the lesions persist for an extended time, secondary cataracts and glaucoma may develop, leading to blindness, which greatly affects the prognosis.

### Fever

Although fever is not included in the triad of symptoms, fever is an important clinical feature of Blau syndrome. Analysis of the clinical manifestations from fifty patients in Japan showed that in about half of the cases (26 out of 50), fever occurred relatively early in the course of the disease (12). Among these patients, ten had intermittent fever, while seventeen had persistent, which includes two patients that exhibited both types. The frequency of the fever was reported to be once every few days to once a month (31, 32), and the duration varied from a few hours to ten days (31, 33). The range of the fever was mostly high (38-40°C) (31, 32, 34–36), with one case of a mild fever (37°C) reported (34).

Fever is important in Blau syndrome diagnosis. De Rose et al. (37) reported that it is essential to keep Blau syndrome in mind as a cause of unknown fever in infants up to four years old. Interestingly, Rosé et al. (38) demonstrated that all three Blau syndrome patients with the *NOD2* p.R587C mutation presented with fever, consistent with our study (12), where eight of nine patients with the p.R587C mutation had fever, compared to four out of 14 patients with p.R334W mutation had fever. In CAPS, there is a clear genotype-phenotype relationship depending on the intensity of the *NLRP3* mutation. However, for *NOD2* mutations, differential enhancement of NF-κB transcription was dependent on the site of the *NOD2* mutation when evaluated in HEK293 cells (15) and the differences did not necessarily correlate with the severity of clinical symptoms in Blau syndrome. Considering that the frequency of cases that present with fever are higher in the p.R587C mutation than in the other mutations, fever may be the clinical symptom that best reflects the ability of NOD2 variants.

## Treatment

Blau syndrome is currently treated with various therapies, with multiple reports on their effectiveness (**Table 1**). However, there is no specific cure for the disease. Currently, the relationship between the variant of the genetic mutation and the response to treatment is not clear.

**Table 1:** Characteristics results of different treatment methods for Blau syndrome.

Treatment	Effective	Not Effective	Side effect
Steroids	• Relieves symptoms (39–42) • Higher doses are required during inflammation, and can be maintained with lower doses during stable phase (40, 43, 44) • Local injection is effective for ocular symptoms (45) • Eye drops is effective for uveitis (43) • Local therapy is effective for uveitis (46) • Effective for skin lesions, joint symptoms, and uveitis (40)	• No effect on uveitis (47) • Uveitis cannot be controlled with steroids alone and requires immunosuppressive agents and biologics (48–50) • Recurrence of ocular symptoms due to steroid reduction (46, 51)	• Growth retardation (48) • Hypertension (39, 48) • Cataract (39) • Diabetes (39) • Avascular necrosis of hip (39) • Iatrogenic Cushing syndrome (51) • Elevated intraocular pressure (51) • Macular edema (52) • Retinal detachment (52)
MTX	• Used with steroids to help reduce steroid use (39, 41, 53) • Effective for skin lesions (43) • Effective for joint symptoms (33) • Effective for mild joint symptoms (42) • Steroid combinations are effective for joint symptoms (47) • Relieves skin and joint symptoms (48) • Effective for uveitis (46) • Good visual prognosis with steroid combinations (54)	• Often needs to be combined with other treatments (51) • Steroid combinations are not effective for joint symptoms (49, 55) • NSAIDs combination is not effective for joint symptoms (55) • No effect on uveitis (47, 50) • Steroid combinations are not effective for uveitis (47, 48, 50, 51) • Steroid combinations are not effective for skin lesions, joint symptoms, and uveitis (48)	
Thalidomide	• Effective for skin lesions, joint symptoms, and uveitis (48)		
NSAIDs	• Effective for pain control on demand (40) • Effective for fever of mild case (42)	• Cannot prevent progression (40) • No effect on joint symptoms (55)	
Cyclosporine	• Effective for uveitis (41)	• No effect on skin lesions, joint symptoms, and uveitis (41)	• Renal dysfunction (41)
Anakinra	• Improves inflammatory symptoms (42) • Normalizes cytokines (42)	• Steroids and cyclosporine combinations are not effective for uveitis (20) • MTX combination is not effective for joint symptoms and uveitis (20)	
Canakinumab	• Effective for severe uveitis (52) • Effective for skin lesions, joint symptoms, uveitis, and fever (56)	• No effect on joint symptoms (56)	
Tofacitinib	• Effective for joint symptoms (55) • Inhibits the production of IL-1, IL-6, IFNγ, and TNF production (55)		
Tocilizumab	• Effective for uveitis (32) • Effective in cases of fever, lymphadenopathy, and hepatosplenomegaly (32)	• No effect on joint symptoms (55, 57) • MTX combination is not effective for joint symptoms (55)	• Anti-tocilizumab antibody appears (57)
Anti-TNF agents	• Effective for joint symptoms (33, 58, 59) • MTX combination is effective for joint symptoms (58)	• Steroid combinations are not effective for skin lesions, joint symptoms, and uveitis (48)	
*Infliximab*	• High quality of life can be achieved with a single agent (41) • Mildly effective for joint symptoms (58) • MTX combination is effective for joint symptoms (33, 58) • Steroid and MTX combinations are effective for joint symptoms (59) • MTX combination is effective for uveitis (60) • Effective for joint symptoms and uveitis (20) • MTX combination is effective for joint symptoms and uveitis (43, 57) • Steroid and MTX combinations are effective for joint symptoms and uveitis (41) • CRP and other laboratory values improve with MTX (57)	• Relapses with MTX reduction/discontinuation (43) • No effect on uveitis (20, 32, 50) • Steroid and MTX combinations are not effective for uveitis (50) • Uveitis relapses (48)	• Fever (48) • Autoimmune encephalitis while treatment with MTX and steroids (61) • Occurs resistance (20)
*Adalimumab*	• Indicated for the treatment of non-infectious uveitis • Effective for joint symptoms (43, 62) • MTX combination is effective for joint symptoms (57) • Effective for uveitis (43, 50, 51, 62) • MTX combination is effective for uveitis (51, 60) • Steroid and MTX combinations are effective for joint symptoms (59) • Steroid and cyclosporine combinations are effective for uveitis (20) • CRP and other laboratory values improve with MTX (57)	• No effect on skin lesions (43) • No effect on joint symptoms (56) • No effect on uveitis (47)	• Facial redness (60)
*Etanercept*	• Effective for joint symptoms (63) • Steroid and MTX combinations are effective for joint symptoms (64)	• Single agent or MTX combination does not work for joint symptoms (20, 41, 63) • NSAIDs combination is not effective for joint symptoms (55) • No effect on uveitis (41) • MTX combination is not effective for skin lesions, joint symptoms, and uveitis (48) • No effect (63)	• Infection (41) • Myelopathy (64)

Other treatments used for Blau syndrome include mofetil mycophenolate (39, 42, 54), azathioprine (11), tacrolimus (12, 39), and cyclophosphamide (39), which are sometimes used in combination.

### Steroids

High doses of steroids can improve clinical manifestations when there is a rapid worsening of joint or ocular symptoms (43, 65); however, prolonged treatment, especially in children, is difficult due to the side effects, including hypertension, growth retardation (48), iatrogenic Cushing syndrome, and elevated intraocular pressure (51). The most common initial diagnosis in our study (12) was JIA with 16 patients. Some of these cases were treated with oral steroids and because these cases were considered relatively mild for JIA, small doses of steroids were continued for an extended time, resulting in a delay in the appropriate therapeutic intervention, and the progression to more severe symptoms, such as joint contractures and blindness.

Topical steroids are often used for skin lesions but are not effective. One patient (12), initially diagnosed with atopic dermatitis, had been treated with topical steroids for an extended time, despite a poor therapeutic response. After a skin biopsy was performed, and granulomas were detected, the patient was subsequently diagnosed with Blau syndrome. Therefore, if a skin rash is not responsive to topical steroids, the possibility of granulomatous diseases, including Blau syndrome, needs to be considered. However, since rashes tends to disappear spontaneously, it can be difficult to evaluate the effectiveness of treatment. In contrast, topical steroid injections and steroid eye drops improved ocular symptoms (45).

### Methotrexate and Other Oral Compounds

Methotrexate (MTX) is effective for joint symptoms (33), and useful for steroid sparing (39), although it often needs to be combined with other treatments (12, 43, 65). In our study (12), excluding the seven patients with no treatment information, out of 43 patients, 25 patients were treated with MTX (seven were in combination with biologics, seven in combination with prednisolone [PSL] and biologics, five in combination with PSL, four with MTX alone, and two in combination with PSL, tacrolimus, and biologics).

Other reports suggest that thalidomide may be effective in the treatment of Blau syndrome (36, 48), but the number of cases is limited, and further studies are needed to evaluate the long-term efficacy and side effects.

In 2018, there was an encouraging report of a significant response to the use of tofacitinib, a Janus kinase inhibitor (JAKi), in patients with cutaneous idiopathic sarcoidosis (66). The patient achieved clinical and histological remission, suggesting that JAK- signal transducer and activation of transcription (STAT) signaling plays a role in the pathogenesis of sarcoidosis and other granuloma diseases. In skin lesion samples taken before treatment, phosphorylated STAT1 (pSTAT1) was identified consistently in granulomas composed of CD68-positive cells, and pSTAT3 was found within inflammatory cells surrounding the lesion; however, these signals disappeared from the granulomas after ten months of treatment with tofacitinib. RNA sequencing results showed that not only interferon-γ (IFNγ), which is dependent on the JAK-STAT pathway, but also tumor necrosis factor-α (TNFα), which is not directly mediated by the JAK-STAT pathway, was elevated in skin lesions before treatment. Expression of these factors was also reduced upon treatment. Based on the successful treatment of sarcoidosis with a JAKi, this treatment has been applied in Blau syndrome and has been reported to be effective. Zhang et al. (55) reported that a single dose of tofacitinib suppressed TNFα and IL-6 production, along with the production of other inflammatory cytokines. Substantial improvements in clinical symptoms and laboratory parameters in the tofacitinib-treated patients were observed.

### Biologics

Interleukin (IL)-1, a downstream product of NOD2 through NF-κB transcription, is anecdotally reported to be elevated in some patients with Blau syndrome, and anti-IL-1β therapy was shown to be effective (52, 56). In general, however, IL-1β cannot be detected in serum from patients with Blau syndrome or in cultures from MDP-stimulated MNC (20, 67). Martin et al. (20) showed that IL-1β is not overexpressed in patients of Blau syndrome and the patients were unresponsive to IL-1β therapy.

Similarly, IL-6, another downstream product of NOD2 activation, increases in some patients with Blau syndrome, and Lu et al. (32) mentioned good responses to tocilizumab, especially for patients with fever, lymphadenopathy, and hepatosplenomegaly. Conversely, Nagakura et al. (57) reported that tocilizumab treatment was discontinued because of recurrent arthritis and the development of anti-tocilizumab IgE antibodies due to the absence of co-therapy with MTX.

### TNF-Targeting Therapy

When the disease is not controlled by steroids or MTX, biologic agents may be useful and anti-TNF agents are primarily used. Infliximab is effective in treating joint symptoms and may prevent the onset of ocular symptoms (68). Adalimumab is indicated for the treatment of non-infectious uveitis and effective for ocular symptoms, joint symptoms, and systemic symptoms (60, 62, 69, 70). However, etanercept-induced myelopathy has been reported in pediatric patients with Blau syndrome (64).

From the patient cohort in Japan (12), 26 out of 43 patients were treated with biologics, all of which were anti-TNF agents (18 patients were treated with adalimumab, five patients with infliximab, two patients with golimumab, and one patient with etanercept). Focusing on the prognosis of ocular symptoms, of the 26 patients that were treated with anti-TNF therapies, only one was blind. In this case, since adalimumab treatment began, the condition in the right eye did not deteriorate. Conversely, of the 14 patients that did not use biologics, five were blind. Of the remaining patients, three were under observation without treatment, seven had unknown treatment details and one was blind. Four patients who had been treated with biologics for JIA prior to their Blau syndrome diagnosis maintained their ocular status despite a relatively advanced age. Thus, we conclude that early treatment targeting TNF is necessary to avoid irreversible ocular symptoms.

## Discussion

An investigation into the cellular phenotypes of Blau syndrome may be necessary to evaluate the efficacy of anti-TNF therapy since the pharmacological mechanism behind their effectiveness in Blau syndrome is unknown.

The IFNγ signal has been reported to be increased in localized skin lesions of idiopathic sarcoidosis and Blau syndrome (71) and IFNγ upregulates the expression of NOD2, acting as a priming signal, as previously reported (22). This was established through iPS cells from patients with Blau syndrome that were differentiated into macrophages and confirmed that IFNγ treatment enhanced the expression of NOD2. The enhanced expression of NOD2 through IFNγ priming was observed regardless of the presence or absence of the NOD2 mutations. However, the spontaneous transcriptional enhancement of NF-κB and the production of proinflammatory cytokines were observed only in cells that contained the NOD2 mutation associated with Blau syndrome. These effects were not found in cells where the NOD2 mutation was corrected to the wild-type amino acid using the CRISPR-Cas9 system. Of interest, skin lesions have appeared following BCG vaccination in some patients with Blau syndrome. Upon review of fifty patients in the cohort from Japan, the attending physician provided information indicating that BCG vaccination may have induced symptoms in nine patients (12). Since IFNγ is a cytokine that is highly associated with BCG-mediated immune responses, BCG vaccination may be involved in NOD2-mediated inflammatory responses through the induction of NOD2 expression.

**Figure 1:**
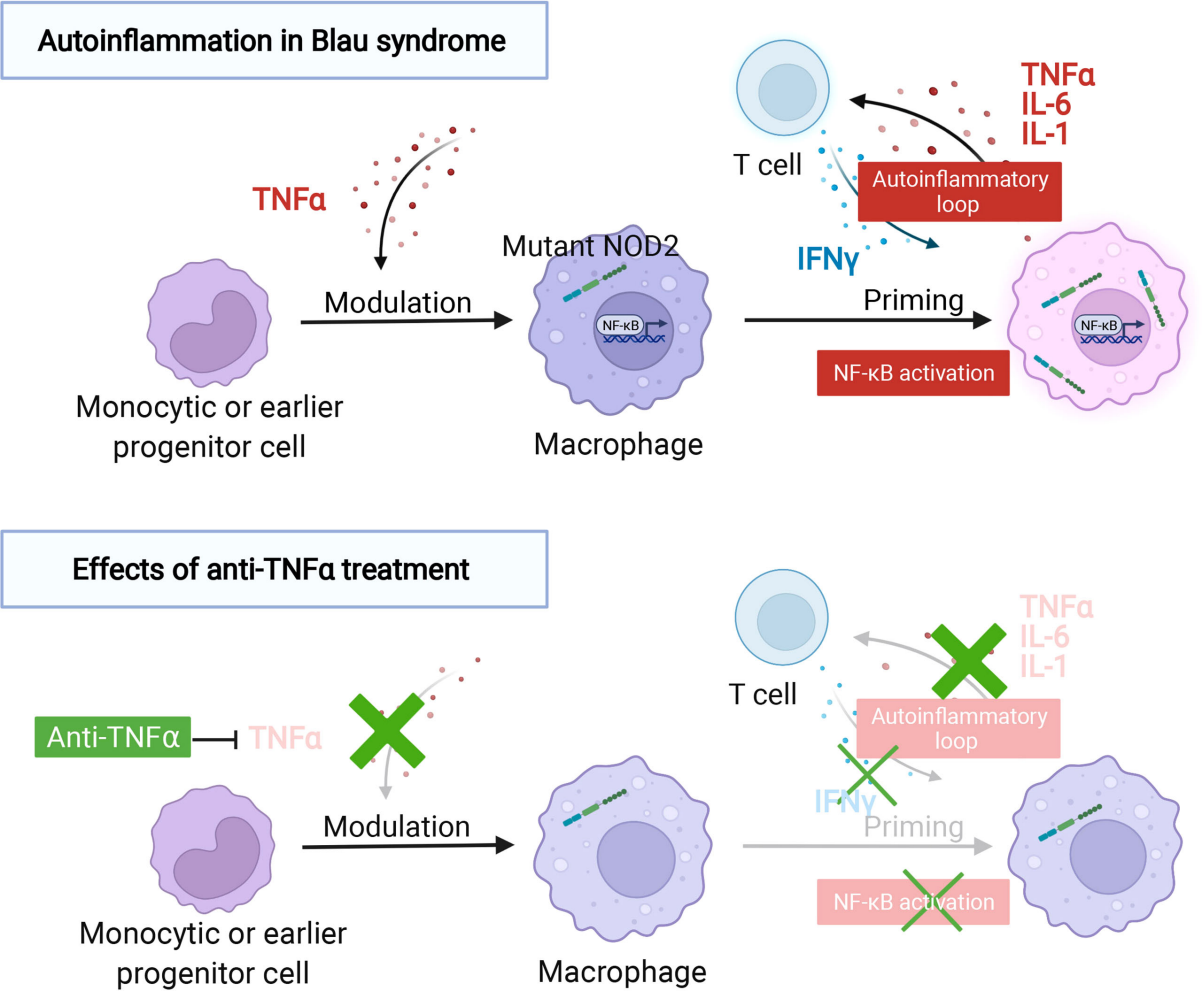
The autoinflammatory response in Blau syndrome and the effect of anti-TNFα treatment. Autoinflammation in Blau syndrome. The expression of NOD2 is induced by IFNγ with or without a NOD2 mutation. The production of proinflammatory cytokines, such as TNFα, IL-6, and IL-1, can only be confirmed in macrophages with NOD2 mutations associated with Blau syndrome, in the absence of muramyl dipeptide (MDP) that activate NOD2. These proinflammatory cytokines further activate T cells and induce the production of IFNγ, thereby establishing a self-amplifying autoinflammatory loop. The effect of anti-TNFα treatment. TNFα-targeted therapy inhibits T cell activation, indirectly suppressing IFNγ production in T cells, and as a result, the autoinflammatory loop is suppressed. Macrophages differentiated from blood collected from patients with Blau syndrome and treated with a TNFα-targeted therapy behaved similarly to wild-type NOD2-expressing cells, despite the presence of the Blau syndrome-associated NOD2 mutations. This suggests that TNFα may exert a modulatory effect on monocytic or early prongenitor cells before they are induced to differentiate into macrophages and adopt a more inflammation-specific form.

While verifying the studies using Blau syndrome patient-derived iPS cells, additional, surprising results were obtained (72). Macrophages, differentiated from the peripheral blood of Blau syndrome who did not receive anti-TNF treatment, released inflammatory cytokines after being primed with IFNγ, as described above. In contrast, macrophages differentiated from patients treated with anti-TNF, did not produce proinflammatory cytokines, similar to macrophages differentiated from healthy subjects. This was despite anti-TNF patient macrophages being primed with IFNγ and being differentiated *ex vivo* for one week with unchanged culture conditions. A comprehensive gene expression analysis of all three groups indicated that the cells from the anti-TNF treatment patient group had a nearly identical gene expression pattern as the cells differentiated from healthy individuals, without the *NOD2* mutation. Furthermore, when primed with IFNγ, cells from these two groups (healthy individuals and anti-TNF treated patients) behaved in the same manner and had gene expression patterns that differed from macrophages differentiated from patients not receiving an anti-TNF agent. This study suggests that prior exposure to a proinflammatory cytokine, such as TNFα, at the monocytic or earlier progenitor cell stage is an important determinant for the macrophage response to IFNγ. Thus, the use of anti-TNF antibodies for Blau syndrome treatment is appropriate in the sense that long-term administration of anti-TNF antibodies may correct the abnormalities that occur in the early progenitor stage by blocking the autoinflammatory loop and restoring the threshold for which IFNγ stimulation triggers an inflammatory response in macrophages (**Figure 1**).

At present, no specific treatment for Blau syndrome exists, based on its etiology, and the disease is treated empirically. However, early diagnosis will allow for the prediction of future symptoms that are likely to appear, and prompt treatment will prevent or delay the onset of severe symptoms (joint contractures and blindness), which significantly impair the patient’s quality of life. Future accumulation of a large number of patients, with detailed information on disease progression, treatment, and prognosis, would be advantageous to analyze disease pathogenesis and establish a specific treatment based on disease etiology.

## Author Contributions

TM, NaK, RT-I, YU, SN, MS, ST, and NoK contributed to conception and design of the study. TM and NaK wrote the first draft of the manuscript. NaK and MS created the first draft of the figure, while RT-I created the final figure. All authors have read, contributed to revisions of the manuscript, and approved the submitted version.

## Funding

This work was supported in part by the Core Center for iPS Cell Research of Research Center Network for Realization of Regenerative Medicine (JP21bm0104001); the Acceleration Program for Intractable Diseases Research Utilizing Disease-Specific iPS Cells (17935423); the Practical Research Project for Rare/Intractable Diseases (17929899) from AMED (MS); a research grant from MHLW (NaK and NoK); a JSPS KAKENHI Grant Number 19K08784, 22K08380; and a Lexi’s Legacy Research Grant from Cure Blau syndrome Foundation (NaK).

## Conflict of Interest

The authors declare that the research was conducted in the absence of any commercial or financial relationships that could be construed as a potential conflict of interest.

## Publisher’s Note

All claims expressed in this article are solely those of the authors and do not necessarily represent those of their affiliated organizations, or those of the publisher, the editors and the reviewers. Any product that may be evaluated in this article, or claim that may be made by its manufacturer, is not guaranteed or endorsed by the publisher.
